# Cross-cultural adaptation of the Taiwan Chinese version of the Falls Efficacy Scale-International for community-dwelling elderly individuals

**DOI:** 10.1186/s12877-022-03597-0

**Published:** 2022-11-19

**Authors:** Kai-Chieh Chang, Hsin-Shui Chen, Yi-Shiung Horng, Horng-Hui Liou, Huey-Wen Liang

**Affiliations:** 1 Department of Neurology, National Taiwan University Hospital Yunlin Branch, Yunlin, Taiwan, ROC; 2 Graduate Institute of Biomedical Electronics and Bioinformatics, National Taiwan University, Taipei, Taiwan, ROC; 3 Department of Physical Medicine and Rehabilitation, National Taiwan University Hospital Yunlin Branch, Yunlin, Taiwan, ROC; 4 Department of Physical Medicine and Rehabilitation, Taipei Tzu Chi Hospital, Buddhist Tzu Chi Medical Foundation, New Taipei City, Taiwan, ROC; 5 Department of Medicine, Tzu Chi University, Hualien, Taiwan, ROC; 6 Department of Neurology and Pharmacology, College of Medicine, National Taiwan University and National Taiwan University Hospital, Taipei, Taiwan, ROC; 7 Department of Physical Medicine and Rehabilitation, National Taiwan University Hospital and College of Medicine, 7, Chong-Shan South Road, 100, Taipei, Taiwan, ROC; 8 Department of Physical Medicine & Rehabilitation, National Taiwan University Hospital Hsin-Chu Branch, Hsin-Chu, Taiwan, ROC

**Keywords:** Balance, Elderly, Fall efficacy, Falls, Fear of falling, FES-I, Taiwan Chinese

## Abstract

**Background:**

The Falls Efficacy Scale-International (FES-I) is a commonly used questionnaire to evaluate concerns about falling. We adapted a Taiwan Chinese version (FES-I_TC_) and evaluated its reliability and validity in community-dwelling elderly individuals. The discriminative validity was tested in relation to several known risk factors for fear of falling (FOF).

**Methods:**

The questionnaire was adapted through translation, back-translation, and expert review processes. A convenience sample of 135 community-dwelling elderly individuals (at least 60 years old) completed the adapted questionnaire, and 31 of them had a retest within 7–10 days. Cronbach’s α and an intraclass correlation coefficient (ICC) were used to evaluate the internal consistency and test–retest reliability. Principal component factor analysis was performed to assess the factor-construct validity. The discriminative validity was tested in relation to demographic features, fall-related history and performances on three functional tests: timed up and go, four-stage balance and 30-s chair stand tests. Effect sizes were computed. Correlation coefficients between physical functional performance and FES-I_TC_ scores were computed. Receiver operating characteristic curves were used to determine the cutoff point for the score to differentiate high and low concern of falling.

**Results:**

The FES-I_TC_ questionnaire had high internal consistency (Cronbach’s α = 0.94) and excellent test–retest reliability (ICC = 0.94). Principal component factor analysis yielded a two-factor model, with several items requiring high demand on postural control loading on factor 2. FES-I_TC_ scores discriminated individuals with different ages, reporting FOF, reporting falls in the past year and using walking aids. However, FES-I_TC_ scores did not differ between the participants who were at risk of falling and those who were not at risk based on functional test performance and there was no correlation found between them.

**Conclusion:**

The FES-I_TC_ was highly reliable and had adequate construct and discriminative validity. The lack of correlation between FES-I_TC_ scores and functional test performance implied the presence of FOF even in individuals with good functional performance. Further follow up studies are warranted to verify the predictive validity of the FES-I_TC_.

## Background

Falls are one of the leading causes of unintentional injuries and deaths among elderly individuals. The rate of any falls ranges between 16.5 and 32.1% in one year and 7.9 and 16.2% in 6 months among community-dwelling elderly individuals according to prospective and retrospective reports [1–6]. Three consecutive national surveys between 2005 and 2013 revealed a one-year prevalence of falls of 16.5 to 21.3% among older adults in Taiwan [6]. The consequences of falls in the elderly population have physical injuries and as well as psychological impacts. Studies have indicated that 22 to 54% of the elderly population has a fear of falling (FOF) after a fall [1, 4, 7]. FOF may reduce the occurrence of falls as individuals avoid relatively dangerous activities, but it can also result in inactivity, social isolation, and potentially deconditioning, functional decline, and decreased quality of life as a consequence [4, 7]. Both FOF and avoidance of activities of daily life (ADLs) predict falls within a year [8]. FOF also plays a significant role in predicting functional recovery after hip fracture surgery, which is more important than the role of pain and depression [9]. Moreover, not only previous fallers experience FOF but also nonfallers experience FOF [10]. A national survey in Korea showed a prevalence rate of FOF up to 96.7% and 75.1% in people with and without a fall history, respectively [11], and one community-based survey showed a prevalence of 53.4% in older adults in northern Taiwan [12]. Therefore, the evaluation of FOF is important for fall risk classification. For example, it is included among the key screening questions of the Stopping Elderly Accidents, Deaths, and Injuries (STEADI) algorithm by the United States' Centers for Disease Control and Prevention (CDC) [13].

The risk factors for FOF are categorized into sociodemographic features, fall-related history, physical function parameters, psychological variables, and clinical examination and history [14]. Several factors have been identified, such as age older than 80 years, female sex, poor perceived general health, multiple falls and limitations in ADLs [7, 15]. A systematic review concluded that the parameters robustly associated with FOF were female sex, performance-based and questionnaire-based physical function and the use of a walking aid [14]. Both previous falls and FOF are important risk factors for falls in the elderly population [9, 16], and these two factors are interrelated, with each as a risk factor for the other [11].

There are different approaches to assess FOF [17]. It can be assessed by a single categorical question [15], or by multi-item questionnaires, such as the Falls Efficacy Scale-International (FES-I) [16], the Fear of Falling Avoidance Behavior Questionnaire [18], the Activities-Specific Balance Confidence Scale [19], and the Geriatric Fear of Falling Measurement [20]. Using a multi-item questionnaire has the advantages over using a single categorical question for covering a comprehensive perspective of FOF in basic and complex daily activities and being sensitive to change during longitudinal studies [16, 21]. These questionnaires are sensitive to discriminating between different levels of fear and assessing FOF with respect to complex physical and social activities [22]. Among these questionnaires, the FES-I is a self-report questionnaire developed by the Prevention of Falls Network Europe (ProFaNE) [16]. It is one of the most widely used tools to assess concerns about falling and asks about the “concerns of falling” in relation to 16 daily activities. It measures the construct of fall-related efficacy, which is the (loss of) confidence in one’s abilities during certain tasks in daily life [17]. The original FES-I has excellent reliability and validity, and by 2022, it has been adapted into more than 35 languages according to the FES-I website. Meanwhile, adaptation of a preexisting measure into different languages should consider the cultural and language differences and use a systematic approach for the process [23]. This process also helps to facilitate cross-study comparison and establish feasibility in different populations. The psychometric properties of the adapted versions of FES-I should also be tested to confirm the maintenance of reliability and validity. Some of the language versions have been validated, including Swedish, Italian, German, Dutch, Turkish, Greek, Portuguese, Brazilian Portuguese, Arabic, Hungarian, Filipino and Persian [24–33]. One Chinese (Cantonese) version is available [34], but use of this version is not feasible in Taiwan because of the different dialects and cultural backgrounds.

The objective of the study was to adapt a Taiwan Chinese FES-I (FES-I_TC_) version of the questionnaire and assess its reliability and validity in a group of community-dwelling elderly individuals.

## Subjects and methods

### Participants and study design

We recruited a convenience sample of elderly individuals with the following inclusion criteria: older than 60 years old, living in the community, speaking Taiwan Chinese and able to walk independently with or without an walking aid for at least 10 m. Individuals were excluded if they had significant cognitive impairment such that they could not follow the instructions, severe visual impairment such that influenced mobility, significant neurological conditions (such as stroke and spinal cord injuries) or major musculoskeletal disorders (such as amputation). This study was approved by the Ethical Committee of the National Taiwan University Hospital (approval number: 202112114RINA, date of approval: 1/14/2022), and written informed consent was obtained prior to participation. The sample size followed the recommendation to include a sample of at least 50 subjects to assess the validity and reliability of an instrument [35]. For the test–retest reliability assessment, we estimated that 30 participants were needed based on an intraclass correlation coefficient (ICC) = 0.5 and two observations per participant with power = 90% and α = 0.05 [36]. For the confirmatory principal factor analysis, we aimed to have at least 100 participants [37].

### Adaptation process

The adaptation was authorized by the ProFaNE and followed the recommended standardized 10-step translation process. The questionnaire contained 16 items scored on a four-point scale (1 = not at all concerned to 4 = very concerned), providing a total score ranging from 16 (absence of concern) to 64 (extreme concern). Two native Taiwan Chinese speakers independently forward translated the original English version into Taiwan Chinese independently. The translators were medical professionals, proficient in English and familiar with the concept of FOF. A combined version was obtained after a meeting to resolve any discrepancies. They also had a personal communication with the original FES-I’s construction team to clarify any minor misunderstandings. This pilot version was given to 10 seniors to confirm its comprehensibility and clarify any ambiguity. Afterward, the translators had a discussion to modify the wording according to the feedback if needed. Then a back-translation was performed by a professional and bilingual English translator who was naive to the purpose and outcome of the study. Finally, all the translators had a consensus meeting to review the back-translation and compared it with the original English version to confirm semantic, conceptual and experiential equivalence.

### Study procedures

All the participants were interviewed to answer the FES-I questionnaire and a questionnaire about the demographic data, fall-related history and one dichotomous question for FOF (yes or no). The demographic data included age, sex, body weight, body height, living condition, and use of walking aids. Then all the participants performed three mobility and dynamic balance tests in random order: the timed up and go (TUG) test [38], the 4-stage balance test (4SBT) [13] and the 30-s chair stand test (30sCST) [39]. These three tests were used to assess the gait, strength and balance of the individuals if they were classified as being at risk of falls according to screening questions included in the CDC’s STEADI algorithm [13]. The tests were performed according to established guidance by two senior physical therapists with at least 10 years of clinical experience.

The convergent validity was tested for the discriminative ability of its scores according to several potential risk factors for FOF, including demographic features, fall-related history and physical function performance test outcomes. A subsample of the 31 participants answered the FES-I_TC_ again within 7–10 days of the initial testing to assess the test–retest reliability of the FES-I_TC._

### Physical functional tests

1. TUG test [38]: The participants stood up from an armed chair, walked at a safe and comfortable pace to a line 3 m away, crossed the line, turned, and returned to a sitting position in the chair. The time required to complete the test was recorded in seconds by a stopwatch. A test result of 12 s or more was categorized as being at risk of falls [13].
2. 4SBT [13]: The participants consecutively performed four standing positions: with feet side-by-side, with the instep of one foot touching the big toe of the other foot (semitandem), with one foot in front of the other and the heel touching the toe (tandem), and on one foot. Each position was described and demonstrated to the patient first. Then the tester stood next to the participants, held their arm, helped them assume the correct position and let go when they were steady. The time they could maintain the position was measured with a stopwatch (recorded in seconds), with the maximal time as 10 s. An older adult who could not hold the tandem stand for at least 10 s was classified as being at risk of falling [13]. The time the participants could maintain for each posture was also summed to obtain a total score.
3. 30sCST [39]: The participants stood up completely from a sitting position on an unarmed and straight back chair (seat height: 45 cm) and then completely back down until they were completely on the seat. The maximum number of chair-stand repetitions completed in a 30-s period was recorded. The participants had a practice of two slow-paced repetitions before formal testing to ensure understanding. The results of the 30sCST were compared against the normative performance data based on age and sex and classified as at risk of falls or not accordingly [13, 40].

### Statistical analyses

The data were tested for normal distribution using the Shapiro–Wilk test and presented and analyzed with parametric or nonparametric tests accordingly. The psychometric properties of the FES-I_TC_ questionnaire were analyzed as follows [35]:

1. The distribution of scores was checked using the mean, standard deviation, median, interquartile range (IQR) and skewness. The proportion of participants who achieved the lowest or highest possible score was computed. More than 15% of participants having the maximal or minimal score was defined as having a ceiling and floor effect, respectively.
2. Test–retest reliability was computed between scores obtained in the first and follow-up survey with ICC estimates and their 95% confidence intervals based on a single rating, absolute-agreement, 2-way mixed-effects model for each condition. An ICC higher than 0.75 was considered excellent, between 0.6 and 0.74 was considered good, between 0.4 and 0.59 was considered fair and less than 0.4 was considered poor [41].
3. The internal reliability of the FES-I was evaluated by calculating Cronbach’s α coefficient for the whole scale. Values between 0.70 and 0.80 were considered a demonstration of good internal consistency, whereas values above 0.80 were considered very good [35].
4. A confirmatory principal factor analysis was performed on the 16 items to examine the internal structure proposed by the original authors and to analyze the factor loading of each item. Varimax rotation and then oblique rotation were used to assess the intercorrelation between factors.
5. Convergent and discriminative validity was examined by computing the between-group difference in total scores with independent *t* test or Mann–Whitney U test. Groupings were based on demographic data (age, sex, use of walking aids, and living alone), single-item reporting of FOF, fall-related history (previous fall history) and the physical functional test results. The age grouping was based on a cutoff value of 80 years according to previous studies [7, 22]. Meanwhile, grouping of the physical functional test scores was performed according to the age- and sex-specific normative performance data as mentioned earlier in the Methods. The effect size (ES) was computed by subtracting the mean scores of two categories for each of variable and dividing by the pooled standard deviation of the two categories. An ES of 0.20 was considered small, 0.50 was considered as medium, and 0.80 was considered large [42]. We also computed the correlation between FES-I scores and scores on three functional tests with Spearman’s *ρ* correlation or Pearson’s correlation, depending on the distribution of the data. The size of the correlation coefficient was interpreted as being very high (> 0.90), high (0.7 to 0.9), moderate (0.5 to 0.7) or low (0.3 to 0.5) [43].
6. Receiver operating characteristic (ROC) curves were used to compute the area under the curve (AUC), sensitivity and specificity to assess a cutoff point according to the single-item question of FOF. An AUC of 0.9 and higher was considered outstanding discrimination, 0.8 to 0.9 was considered excellent discrimination and 0.7 to 0.8 was considered acceptable discrimination [44].

Statistical analyses were performed using SPSS 15.0 for Windows (SPSS Inc., Chicago, USA) with a level of statistical significance set at *p* < 0.05.

## Results

One hundred and thirty-five participants completed the questionnaires and three functional tests. Their mean age was 71.1 ± 7.2 years, and 71% were women (Table 1). The proportions of single falls and recurrent falls in the past year were 14.1% and 6.7% respectively. The total FES-I_TC_ scores ranged from 16 to 58, with a mean, median and skewness of 26.2, 23.0 and 1.2, respectively. None of the participants had the highest score and 14.8% of them had the lowest scores, demonstrating no ceiling or floor effects. The FES-I_TC_ and functional test scores violated a normal distribution, so nonparametric methods were used for data analysis.

**Table 1:** Demographic data of the participants presented as the mean ± standard deviation or number and percentage

Variables	Results
Age (years)	71.1 ± 7.2
Female	96 (71.1%)
BMI (kg/m^2^)	24.7 ± 4.1
Education level	
Elementary school or less	62 (45.9%)
Secondary school	42 (31.1%)
College or above	31 (23.0%)
Living alone	36 (26.7%)
Using walking device for outdoor activities	8 (5.9%)
Fall history in the past one year	
No	107 (79.3%)
Once	19 (14.1%)
More than once	9 (6.7%)

The Cronbach’s α coefficient of the FES-I_TC_ was 0.94, demonstrating very good internal consistency. The interitem correlation was an average of 0.52 (range 0.31–0.76). Item 11 (walking on a slippery surface) had the highest mean score followed by item 14 (walking on an uneven surface) and item 15 (walking up or down a slope) (Table 2). Thirty-one of the participants had a retest in 7–10 days, and the ICC_(2,1)_ for the total score was 0.94 (95% CI: 0.88–0.97, *p* < 0.001). Principal component analysis (Kaiser–Meyer–Olkin test = 0.93, Bartlett’s test of sphericity; *p* < 0.001) with varimax rotation yielded 2 factors with eigenvalues > 1, explaining 62.5% of the total variance. For the two-factor model, factor 2 was dominated with items assessing concern about the more demanding balance control activities: 9 (reaching up or bending down), 11 (walking on a slippery surface), 14 (walking on an uneven surface), 15 (walking up or down a slope) and 16 (going out to a social event) (Table 2). The Cronbach’s α coefficient of each of the two factors was above 0.9.

**Table 2:** Descriptive data of each item of the FES-I and the results of principal component analysis

Items	Mean ± Standard deviation	Median	Two-factor solution		Communality
			Factor 1 loading	Factor 2 loading	
1.Cleaning the house	1.7 ± 0.8	1	0.45	0.47	0.42
2. Getting dressed/undressed	1.4 ± 0.7	1	0.67	0.35	0.58
3. Preparing simple meals	1.3 ± 0.5	1	0.80	0.19	0.67
4. Taking a bath or shower	1.7 ± 0.9	1	0.51	0.46	0.47
5. Going to the shop	1.4 ± 0.7	1	0.77	0.27	0.67
6. Getting in or out of a chair	1.5 ± 0.8	1	0.73	0.39	0.68
7. Going up or down stairs	1.8 ± 0.8	2	0.52	0.43	0.46
8. Walking around outside	1.4 ± 0.7	1	0.66	0.43	0.62
9. Reaching up or bending down	1. 0 ± 0.9	2	0.30	0.81	0.75
10. Answering the telephone	1.4 ± 0.8	1	0.61	0.33	0.48
11. Walking on a slippery surface	2.2 ± 1.0	2	0.25	0.84	0.77
12. Visiting a friend/relative	1.3 ± 0.6	1	0.81	0.23	0.71
13. Going to a place with crowds	1.6 ± 0.8	1	0.67	0.44	0.65
14. Walking on an uneven surface	2.1 ± 0.9	2	0.38	0.75	0.71
15. Walking up or down a slope	2.0 ± 0.9	2	0.27	0.80	0.71
16. Going out to a social event	1.6 ± 0.9	1	0.48	0.65	0.65
Eigenvalues			8.8	1.2	
Explained variance			55.0%	7.5%	62.5%
Cronbach’s α			0.92	0.90	

Approximately half of the participants reported FOF according to the single dichotomous question, and the proportions among nonfallers, single-fallers and multiple-fallers were 48.6%, 31.6% and 100%, respectively. The FES-I_TC_ scores were significantly higher in participants using walking aids (*p* < 0.001), having FOF (*p* < 0.001), with a history of falling (*p* < 0.001), and 80 years or older (Table 3). The ES was the largest for using walking aids (1.63), having multiple falls (1.51) and being at risk based on the 4SBT (1.12) (Table 3). However, there was no significant difference between men and women, living alone or not, and groups classified by the performance test results. Moreover, FES-I_TC_ scores had a low correlation with each of the three functional test results, with Spearman *ρ* correlation coefficients as 0.18 for the TUG (*p* = 0.04), -0.33 for the 4SBT (*p* < 0.001) and -0.28 for the 30sCST (*p* = 0.001).

**Table 3:** Between-group difference in the FES-I scores

Variable	N (%)	Mean ± standard deviation	Median (IQR)	*P* value	Effect size
All participants	135	26.0 ± 9.5	23 (13)		
Demographic characteristics					
Age				0.02	0.73
< 80 years old	116 (86.0%)	25.1 ± 8.5	23 (12)		
≥ 80 years old	19 (14.4%)	32.0 ± 12.5	31 (22)		
Sex				0.10	0.18
Men	39 (28.9%)	24.8 ± 10.2	22 (12)		
Women	96 (71.1%)	26.5 ± 9.1	24 (12)		
Use of walking aids				< 0.001	1.63
No	127 (94.1%)	25.1 ± 8.8	23 (12)		
Yes	8 (5.9%)	40.5 ± 8.7	37.5 (13.3)		
Living alone				0.20	0.28
No	99 (73.3%)	25.3 ± 9.0	23 (12)		
Yes	36 (26.7%)	28.0 ± 10.6	28 (16.3)		
Falling risk factors					
Falls in the past one year				< 0.001	0.53
None	107 (79.3%)	25.0 ± 9.1	23 (13)		
Any falls	28 (20.7%)	30.0 ± 10.0	29 (14.8)		
Multiple falls in the past one year				< 0.001	1.51
No	126 (93.3%)	25.1 ± 8.7	23 (12)		
Yes	9 (6.7%)	39.4 ± 9.3	38 (16)		
Fear of fall				< 0.001	0.87
No	68 (50.4%)	22.2 ± 8.1	18.5 (9.8)		
Yes	67 (49.6%)	30.3 ± 9.2	29 (12)		
Physical functional tests					
Timed up and go				0.67	0.32
No risk	126 (93.3%)	25.8 ± 9.2	23 (12)		
At risk of falling	9 (6.7%)	28.9 ± 12.9	31 (22)		
4-stage balance test				0.09	1.12
No risk	130 (94.8%)	25.6 ± 9.1	23 (12)		
At risk of falling	5 (5.2%)	36.2 ± 14.2	35 (26)		
30 s chair stand test				0.76	0.003
No risk	126 (93.3%)	26.0 ± 9.6	23 (13)		
At risk of falling	9 (6.7%)	26.0 ± 7.7	25 (13)		

The ROC analysis showed that the AUC for the FES-I_TC_ was 0.79 for having FOF (*p* < 0.001), with a cutoff score of 20.5 to differentiate between participants with low and high levels of concerns. The sensitivity and specificity were 0.93 and 0.57, respectively.

## Discussion

We adapted a Taiwan Chinese version of the FES-I (FES-I_TC_) in community-dwelling elderly individuals. Its reliability was consistent with the original version [16], with high internal consistency and a high ICC for test–retest reliability. It also demonstrated good discriminative ability with several demographic features, fall-related history and self-reported FOF. However, we were not able to document a correlation between FES-I_TC_ scores and scores on three physical functional tests. The cutoff score to differentiate those with high or low levels of concern for falling was 20.5, similar to the cutoff value for low (16-19) vs. moderate and high concerns (20 and more) in the original version^21^. The study provided an additional language version of the FES-I to assess concerns about falling during daily activities. The low correlations of the physical functional test scores and FES-I scores also highlighted the high prevalence of FOF in community-dwelling elderly individuals with good physical function. The similarities and discrepancies between our study and previous studies warrant further discussion.

The mean and median scores of the FES-I_TC_ in the current study were 26 and 23, which fell within a previously reported range of mean values between 20 and 36 points among community-dwelling elderly individuals [22, 32, 34, 45, 46]. Similar to some observations, we found that the distribution of scores showed a skewness toward assessing people with higher levels of concern about falling [21]. Nearly 15% of our participants reported the lowest score. This result raised concerns about the ability of the FES-I to track the full range of concern about falling among healthy older people and a potential lack of responsiveness to change among nonfrail older community-dwelling adults [11]. Some researchers have suggested adding more demanding balance-related activities [21].

Meanwhile, it was noteworthy that the reported fall history in the past year of our participants was 14.1% for any falls and 6.7% for multiple falls, but up to half of them reported FOF. Comparatively, the proportion of reporting any FOF ranged from 62.3 to 72% with a 4-point Likert scale in several validation studies [22, 32, 45]. The range of community-dwelling elderly individuals reporting FOF is wide in epidemiological studies, mostly between 20 and 80% [47]. The wide range of differences was attributed to the selection of participants, the use of different responses and different cultural behaviors toward FOF [22].

The reliability was confirmed for the adapted FES-I_TC_ questionnaire. The test–retest reliability of the adapted version within 7–10 days was high, with an ICC of 0.94. This result was consistent with most previous results, with an ICC of approximately 0.95 within 7–10 days [16, 28], 0.79–0.93 at 2 weeks [31, 32, 45], and 0.79–0.82 within four weeks [22, 48]. The internal consistency evaluated by Cronbach’s α was also high (0.94), which was within the previously reported range; that was, mostly between 0.90 and 0.98 [16, 22, 28, 45, 49]. However, high Cronbach’s α and interitem correlation values imply that the FES-I could be shortened. Several items (items 1, 2, 3, 4, 6, 8, 10 and 16) assessed high levels of concern in a small distribution, suggesting some redundancy among these items [22]. A short version of the FES-I with 7 items (items 2, 4, 6, 7, 9, 15 and 16) has been developed [48]. It has a slightly lower Cronbach’s α coefficient than the original version (0.92 vs. 0.96), but similar reliability and discriminative ability. However, the short version has a higher floor score than the original version, even among Parkinson’s disease or hospitalized patients [50, 51]. Further studies are warranted to examine the psychometric properties of an adapted short version.

We used a confirmatory principal analysis to provide a construct analysis and fit a two-factor model, which explained 62.5% of the variance. The results were similar to the original FES-I and supported the construct validity. For our two-factor model, factor 2 included items assessing concerns about activities that demand higher balance control (items 9, 11, 14, 15 and 16), and the results were partly consistent with other studies. For example, the items loading on factor 2 were items 8, 9, 11, 13, 14, and 15 in the original version [16], items 4, 9, 11, 14, and 15 in the Norwegian version, items 4, 11, and 14 in the Turkish version [52], and items 4, 7, 11, 14, and 15 in the Hungarian version [31]. The slight differences among studies have been explained by variation in the cultures, environments, or tested populations.

The discriminative validity of the FES-I is often tested according to known factors for FOF, including demographic features, fall history, fear of falls, fall risks, medical conditions, physical performance, quality of life, and psychological factors [22, 32, 45, 52, 53]. We documented significantly higher FES-I_TC_ scores in the individuals 80 years and older, reporting FOF, having previous falls or multiple falls and using walking aids. These variables have been identified as risk factors for FOF [14], and the results supported the convergent validity of the FES-I_TC_. The ES was the largest for those using walking aids (1.63), followed by those having a history of multiple falls (1.51). The self-reported FOF also had a large ES, while age and any fall in the past year each had a medium ES. Meanwhile, we did not find between-group differences regarding to sex and living alone as reported by some other studies, although the FES-I_TC_ scores were slightly higher in women versus men and in individuals living alone than in those living with family.

Physical functional tests have been used to test the convergent validity of the FES-I, with the assumption that increased concerns of falling are correlated with reduced physical function performance. These performance tests aim to assess mobility function, with the TUG most frequently used [28, 30–32, 45, 52, 53]. Other studies have included the Berg balance scale and gait speed [32]. We selected three physical function tests that have been recommended to test for gait, strength or balance problems when possible fall risks were identified by screening questions according to the CDC’s STEADI algorithm [13]. The associations between functional test scores and the FES-I_TC_ scores were assessed with two approaches: comparing the between-group difference with preset criteria for being at risk or not according to the test scores, and computing correlation coefficients between the test results and FES-I_TC_ scores. For the first approach, a large ES was observed with the 4SBT, but the FES-I_TC_ scores were not different between the participants classified as being at risk of falling and not at risk in all three tests. With correlation analysis, the Spearman’s ρ correlation coefficients between FES-I_TC_ scores and scores on each functional test were all lower than 0.3, indicating no correlation. The results were not in accordance with studies assessing versions in several other languages, which showed moderate to high correlations between FES-I and TUG scores (*r* = 0.5–0.74) [28, 30, 31, 52, 53]. However, some other studies had results similar to our study. In the assessment of the Filipino version, there was a poor correlation between FES-I and TUG scores (*r* = 0.25) and FES-I scores failed to discriminate the participants with or without fall risk classified by the short-form Physiological Profile Assessment [32]. One explanation was the good balance function of the current sample of community-dwelling elderly individuals who were generally independent and ambulatory. Only 6% of them required walking aids for outdoor activities and less than 10% of them were classified as being at risk of falling on scores on the three physical function tests. This was apparently lower than the proportion based on different criteria in some other studies, for example, using 15 s for classification resulting from the TUG test [34]. The low correlation between functional performance scores and FES-I scores also implied that high concerns for falls might precede declines in balance function in elderly individuals.

We used the reporting of FOF to establish the cutoff point of the FES-I with ROC analysis. With a cutoff point of 20.5, the sensitivity and specificity were 0.93 and 0.57, respectively. In comparison, the official website of the FES-I suggests using a score of 16 to 19 to indicate low concerns, 20 to 27 to indicate moderate concerns and 28 to 64 to indicate high concerns according to a longitudinal validity study [21]. The AUC with this cutoff point was similar to other versions, ranging from 0.70 to 0.81 [52], although several versions of the test in other languages reported a slightly higher cutoff point at 22 to 24 [32, 52]. The high sensitivity and relatively low specificity in our study suggested that the FES-I_TC_ scores with this cutoff point could correctly identify those with FOF, but were less likely to correctly identify those without FOF. With more studies across different cultures and settings, these cutoff points could help to establish norms for acceptable ranges of FOF in clinical practice and clinical trials. Meanwhile, the cutoff scores must be understood and used with caution, since the establishment of the cutoff point could be based on different endpoints, populations and settings.

### Study limitations

There were two limitations that should be addressed. The participants were mostly independent and ambulatory community-dwelling individuals. There was a high proportion of participants with floor scores on the FES-I_TC_ but no participants with ceiling scores. This assessment should be tested in populations with a higher risk of falls to evaluate possible ceiling effects. Second, we did not evaluate responsiveness, which is an important psychometric property for longitudinal studies and when using this as an outcome measure for intervention studies. Longitudinal studies would help to clarify this psychometric property.

## Conclusion

This adapted FES-I_TC_ had satisfactory reliability and validity to facilitate comparison between studies and populations in different countries and settings. Our results highlight a high prevalence of FOF among healthy community-dwelling elderly individuals who had generally good functional test performance. Since FOF is an important risk factor for falls, screening of FOF is an important assessment in this population.

## Data Availability

The datasets generated and/or analyzed during the current study are not publicly available due to the restriction under the institutional ethical committee ‘s policy, but may be available from the corresponding author on reasonable request and with permission of the ethical committee.

## Abbreviations

4SBT: 4-Stage balance test
30sCST: 30-Second chair stand test
AUC: Area under the curve
CDC: Centers for Disease Control and Prevention
ES: Effect size
FES-I: Falls Efficacy Scale-International
FES-I_TC_: Taiwan Chinese version of Falls Efficacy Scale-International
FOF: Fear of falling
ICC: Intraclass correlation coefficient
IQR: Interquartile range
ms: Milliseconds
ProFaNE: Prevention of Falls Network Europe
ROC: Receiver operating characteristic
STEADI: Stopping Elderly Accidents, Deaths, and Injuries
TUG: Timed up and go
